# Split spinal cord malformations in 4 Holstein Friesian calves

**DOI:** 10.1186/s12917-019-2055-x

**Published:** 2019-08-28

**Authors:** Lara Górriz-Martín, Jasmin Neßler, Iris Voelker, Sina Reinartz, Andrea Tipold, Ottmar Distl, Andreas Beineke, Juergen Rehage, Maike Heppelmann

**Affiliations:** 1 Clinic for Cattle, University of Veterinary Medicine Hannover, Hanover, Germany; 2 Department Small Animal Medicine and Surgery, University of Veterinary Medicine Hannover, Hanover, Germany; 3 Institute for Pathology, University of Veterinary Medicine Hannover, Hanover, Germany; 4 Institute for Animal Breeding and Genetics, University of Veterinary Medicine Hannover, Hanover, Germany

**Keywords:** Calf, Split spinal cord malformation, Diplomyelia, Diastematomyelia

## Abstract

**Background:**

The split spinal cord malformation (SSCM) is an uncommon congenital malformation of the vertebral canal in which parts of the spinal cord are longitudinally duplicated. In SSCM Type I, each spinal cord has its own dura tube. In the SSCM Type II, both parts of the spinal cord are surrounded by a common dura tube.

**Cases presentation:**

During the clinical examination one calf showed ambulatory paresis and 3 calves non-ambulatory paraparesis. Calf 4 additionally had a congenital tremor. The examination of calf 4 using magnetic resonance imaging (MRI) showed a median hydrosyringomyelia at the level of the 4th lumbar vertebra. The caudal part of this liquid-filled cavity was split longitudinally through a thin septum. From there, the spinal cord structures duplicated with an incomplete division, so that the transverse section of the spinal cord appeared peanut-shaped and in each half a central canal could be observed. The pathological-anatomical examination after euthanasia showed a duplication of the spinal cord in the area of the lumbar vertebral column in all calves. The histopathological examination revealed two central lumbar vertebral column channels. The two spinal cord duplicates were each surrounded by two separate meninges in calf 2 (SSCM type I); in the other calves (1, 3, 4, and) the two central canals and the spinal cord were covered by a common meninx (SSCM type II). A pedigree analysis of calves 2, 3 and 4 showed a degree of relationship suggestive of a hereditary component. This supports the hypothesis of a possible recessive inheritance due to common ancestors, leading to partial genetic homozygosity.

**Conclusions:**

The clinical appearance of SSCM can vary widely. In calves with congenital paralysis SSCM should always be considered as a differential diagnosis. A reliable diagnosis intra vitam is possible only with laborious imaging procedures such as MRI. Further studies on the heritability of this malformation are necessary to confirm a genetic cause of this disease.

## Background

Several congenital abnormalities of the vertebral column and spinal cord are referred to as spinal dysraphisms [1, 2]. Diastematomyelia and diplomyelia are terms traditionally used to describe the two main forms of a dysraphism characterized by the longitudinal division of segments or the whole spinal cord, cauda equina, and filum terminale by a dorsal septum [2, 3]. However, these two terms have often been used inconsistently in pediatrics, neurology and veterinary medicine, leading to ambiguities in the diagnosis, treatment and prognosis, since their embryogenesis differs [4, 5]. Pang et al. [4] and Dias and Pang [6] proposed a classification of these two abnormalities based upon the composition of the dural sheath and the implicated mesenchymal tissue. Whereas a Type I split spinal cord malformation (SSCM) consists of two hemicords with dural tubes and separation by dura-sheathed rigid osseo- or fibrocartilaginous median septum, the hemicords of Type II SSCM are encased in a single dural sac and only separated by a nonrigid, fibrous median band.

The exact embryological mechanisms leading to SSCM have not yet been clarified. The most accepted theory [4] is based on the existence and persistence of an abnormal accessory neurenteric canal [7] as a result of adhesions between ecto- and endoderm.

Spinal cord malformations represent a sporadic pathology in calves compared with other animal species, where they are rare [5, 7–12]. Reports of spinal cord duplication in cattle date from 1926 [13] and cases of SSCM Type II (diplomyelia) are more common than Type I (diastematomyelia) [14]. Both genders are susceptible [7, 8, 10, 15–17]. Calves are presented to the veterinarian shortly after birth. Exceptionally, one case reported a 7.5-month old calf [17]. The breeds in which SSCM was diagnosed were: Hereford [18], Holstein Friesian [8, 10], Japanese Black [16, 17], and Holstein x Belgian Blue crossbred [15].

Cases were usually presented due to abnormal posture, abnormal gait or inability to stand [7, 14–17]. Patients with SSCM are often ambulatory but usually present spastic paraparesis up to -plegia and proprioceptive ataxia of the pelvic limbs without signs of decreased consciousness or cranial nerve deficits. A thoracolumbar kyphosis and sometimes a rotation in the vertebral column might be visible. The gait is very characteristic with hypoflexion in all joints with protraction of both pelvic limbs at one time referred to as “bunny hopping” [7, 14, 15]. Depending on the location and extension of the lesion the limb withdrawal reflex, patellar reflexes, superficial and deep sensitivity responses, and nociception may be delayed, and cutaneous trunci reflex, perineal, sphincter and anal tone may be normal [7, 15]. Deficits in urination or defecation are not specified in cattle but humans affected by SSCM Type I showed bowel and urinary incontinence [19]. The palpation of the vertebral column seems not to trigger a pain reaction [15]. In some cases a meningomyelocele (cutaneous structure with/without hypertrichosis) may be present overlaying the anatomical area of the SSCM lesion and may contain cerebrospinal fluid [7]. Cerebrospinal fluid examination is normal [7]. Whereas a SSCM might not always be detected by means of radiography and myelography, ultrasonography are suitable diagnostic tools in calves [7, 15, 17]. The anatomical location preferred for sonography is the lumbosacral junction (L6-S1; [15]). Magnetic resonance imaging (MRI) is the gold-standard for in vivo diagnosis [7]. This technique permits the detection of a SSCM (type and extension) in any location of the spinal cord, and concomitant pathologies can be evaluated [3, 7]. The macroscopical findings at autopsy might vary but the spinal cord appears duplicated, most frequently at the lumbar level but other levels such as the cervical intumescence [7] may be affected [15–17]. The classification into SSCM Type I or II is performed using histopathology.

The current case series describes clinical (calf 3 and 4) and imaging (calf 4) findings of two Holstein-Friesian calves diagnosed with SSCM Type II. Additionally, histopathological findings of two further Holstein-Friesian calves affected by SSCM Type I (calf 1) and II (calf 2) are also reported as well as the pedigree analysis of 3 calves (calves 2, 3 and 4).

## Cases presentation

### Signalment / history

The four calves with difficulty or inability to stand were hospitalized in the Clinic for Cattle of the Veterinary University of Hannover between 1999 and 2015: Through subsequent diagnostic examinations SSCM was diagnosed in all the cases. The animals were allocated in cubicles (approx. 5 m^2^) provided with rubber mats on the floor and plenty of straw. These cubicles are set aside for bovine patients under 100 kg of body weight and are thoroughly disinfected after their medical discharge. The patients had free access to water, hay and calf corn in the cubicle. Two liters of whole milk were provided 4 times daily with nipple bucket or feeding bottle depending on the ability of the calf to stand. Body temperature was measured twice a day (at 06:30 a.m. and at 05:30 p.m.) and a clinical examination was performed at least once a day.

**Case 1:** 19-day old, male, Holstein Friesian. The calf was hospitalized in July 1999 because of inability to stand on his own since birth and a prominent scoliosis.

**Case 2:** 24-day old, female, Holstein Friesian. The calf was hospitalized in November 2002 because of ambulatory paraparesis since birth.

**Case 3:** 9-day old, female, Holstein Friesian. The calf was hospitalized in July 2012 because of suspected trauma of the femoral nerve after a distocic delivery with paraparesis.

**Case 4:** 9-days old, male, Holstein Friesian. The calf was hospitalized in June 2015 because of permanent generalized tremor and difficulty to stand.

Only in case 4 the history of dam and sire is known. The dam had already given birth to two normal calves previously. There was no additional maternal history. The sire is widely used in Germany for artificial insemination and the pedigree is known.

### Clinical findings

In cases 1 and 2 the clinical examination was performed but was no longer available at the time when this manuscript was written. In cases 3 and 4 the clinical examination was performed and available.

**Case 3:** The calf was alert, lying in sternal position with extended pelvic limbs to the right, and attempted unsuccessfully to stand. Joints and limbs were anatomically normal. Orthopedic examination of limbs was normal. The animal showed a body temperature of 39.1 °C. The extra-abdominal umbilicus was approx. 3 cm thick with indurate consistency and was sensible to pressure. Deep palpation of the intra-abdominal structures (umbilical vein, arteries and urachus) revealed a physiological regression. Further organ systems (excluding the nervous system) were normal. The animal was treated 4 days with Procaine Penicilline G 300 mg (benzylpenicilline-proaine 1 H_2_O 300 mg; aniMedica, Senden-Bösensell, Germany; 20,000 I.U./kg BW, s.c.).

**Case 4:** The calf was alert, lying in sternal-left-lateral-position, with extended right thoracic and both pelvic limbs. Some areas of the skin (perineum and lateral portion of the left pelvic limb) were devoid of hair without macroscopic cutaneous lesions. A moderate muscle atrophy was present in both pelvic limbs (left > right). During the examination the calf showed a generalized, persistent, low-frequency tremor ( [20]; approx. 1–2 Hz predominantly visible at the head, increasing and spreading to the whole body if excited). The tremor was not present if the animal was completely alone and the environment was silent. Body temperature of the calf was 39.2 °C. Respiratory frequency (68 breaths/min) were increased and the respiratory sounds were bilaterally enhanced in the tracheo-bronchial and bronchio-broncholar areas and a moderate inspiratory dyspnea was present. The extra-abdominal umbilicus had a thickness of approx. 2 cm, indurated consistency, and was sensible to pressure. The surrounding skin was reddish, warm and purulent exudate was present in the distal aspect. Through deep palpation a distal mild enlargement of the umbilical vein was stated. The umbilical arteries and urachus showed a physiological regression. Other organ systems showed physiological findings. In the right carpal joint a superficial decubital lesion was present with a diameter of 1 cm. Under this lesion and in the anatomical area of the bursa precarpalis and the joint capsule a slight fluctuation was detectable. The right carpal joint and the rest of the evaluable joints were clinically normal. During the orthopedic examination the animal was reluctant to fully stand on the thoracic right limb. The animal was treated daily with Duphamox® 150 mg/ml (amoxicilline-trihydrate 172.2 mg; Zoetis, Louvain-la-Neuve, Belgium; 7 mg/kg BW, s.c.). The decubital lesion was treated with Zincojecol® (zincoxide cod liver oil ointment; Garbsen, WDT Germany; topically).

### Neurological findings

**Case 3:** Non-ambulatory paraparesis with physiological muscle tone in all limbs but decreased patellar reflexes in both limbs. Cutaneous trunci reflex was reduced bilaterally. The right pelvic limb was kept flexed and in abduction. Tail and sphincter tonus were normal. No cranial nerve deficits were appreciated and mentation was appropriate. Neuroanatomical localization was L4–6 spinal cord segments.

**Case 4:** Calf was found in sternal recumbency not able to rise. There was an action tremor triggered by excitement and stress as well as an intention tremor. Increased muscle tone of all limbs and a tendency for opisthotonus could be appreciated. When the calf supported it was ambulatory with assistance but showed ataxia in all limbs and spastic tetraparesis which was much more profound and associated with “bunny hopping” in the pelvic limbs. The calf showed a tendency to circle to the right. Proprioception was absent in the pelvic but normal in the thoracic limbs. Spinal reflexes were normal on the thoracic limbs but a slightly decreased patellar reflexes as well as decreased withdrawal reflex were present on both pelvic limbs. Cutaneous trunci reflex was normal. Tail was actively moved. Perineal, sphincter and anal tone were normal. The calf was bright and alert without cranial nerve deficits. Palpation of the vertebral column did not trigger any pain reaction. Neuroanatomical localization was suspected to be multifocal including L4-L6 spinal cord segments and cerebellum or generalized spinal cord white matter tracts. A myelin disorder, such as hypomyelination or demyelination, were also considered as differential diagnosis for the tremor.

### Clinical diagnosis

**Case 1:** Non-ambulatory paraparesis.

**Case 2:** Ambulatory paraparesis.

**Case 3:** Non-ambulatory paraparesis and omphalitis simplex.

**Case 4:** Non-ambulatory paraparesis, congenital tremor, acute bronchopneumonia, omphalophlebitis, bursitis precarpalis.

### Hematological and biochemistry profiles

**Case 3:** A mild leukocytosis (10,800 leukocytes/μL) was detected.

**Case 4**: A slight neutrophilic leukocytosis (15,000 leukocytes/μL; 64% band neutrophils) was detected. Thiamin-monophosphat (2.22 μg/L), thiamin-diphosphat (44 μg/L) concentrations were reduced compared to reference values (> 6.2 μg/L and > 79 μg/L, respectively), but thiamin concentration (3.56 μg/L), and total vitamin B1 (85 μg/L) were normal (> 1 μg/L and > 50 μg/L, respectively). Infectious bovine rhinotracheitis (IBR) was assessed serologically with ELISA and resulted negative. Bovine virus diarrhea (BVD) 1 and 2 were examined with both antigen ELISA and serology ELISA tests and were consistently negative. Detection of Schmallenberg (SBV) virus via real-time PCR was negative.

### Imaging diagnosis (MRI)

**Case 4**: MRI (3.0 T MRI scanner Achieva, Philips Medical Systems, Best, The Netherlands; spinal coil) was performed under general anesthesia (Xylavet® (xylazine hydrochloride, 20 mg/ml; CP-Pharma Handels GmbH, Burgdorf, Germany; 0.1 mg/kg, i.v.) in combination with Ursotamin® (ketaminehydrochloride 100 mg/ml; Serumwerk Bernburg AG, Bernburg, Germany; 2 mg/kg, i.v.) in supine position. T1w and T2w sequences in sagittal planes were generated of the skull and the whole vertebral column up to the sacrum while transversal planes were taken from L2 to the sacrum. The extended MR Workspace V2.6.3.5 was used for analysis (Philips Medical Systems, Best, The Netherlands).

**Fig. 1:**
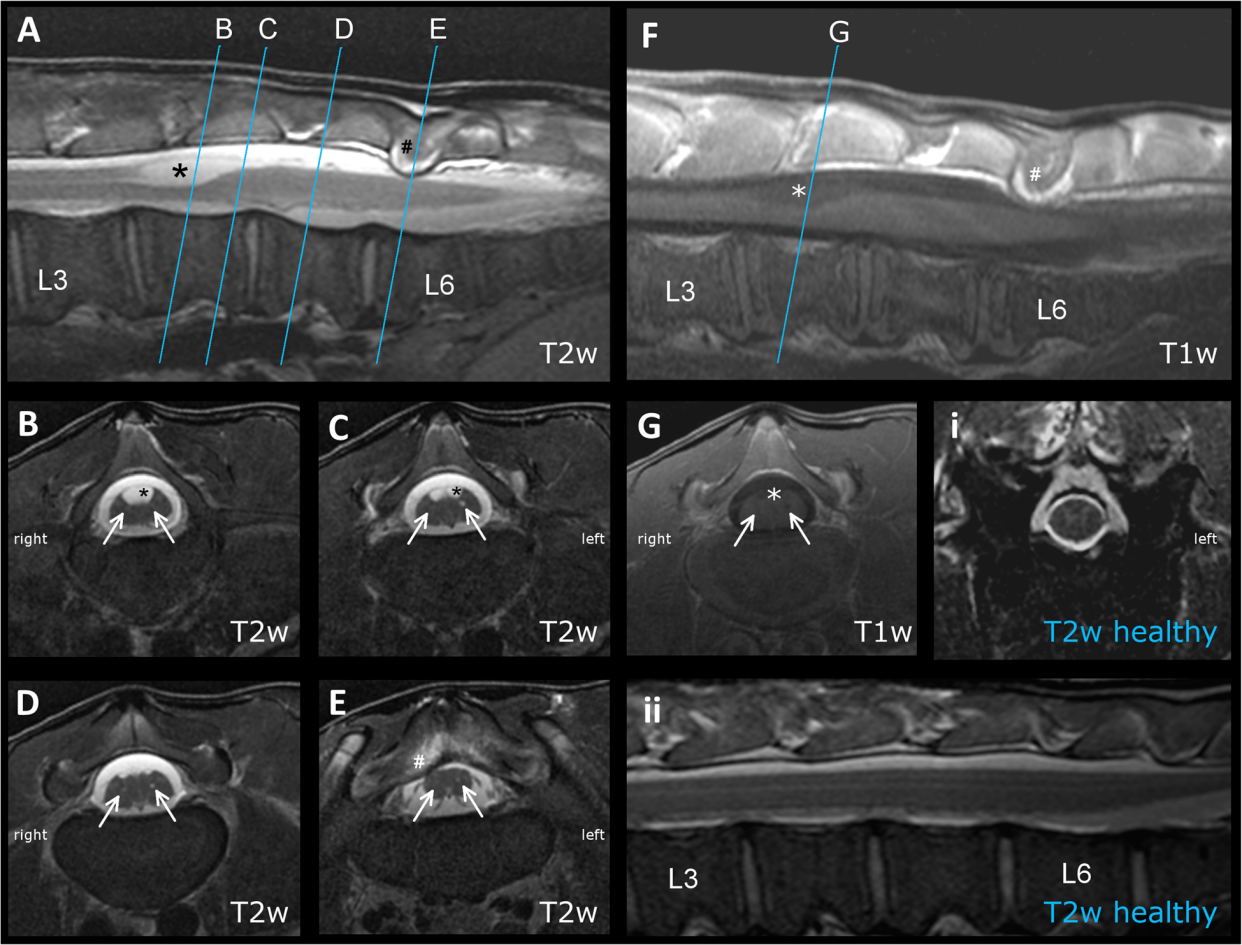
Magnetic resonance imaging (MRI) of a calf with SSCM type II (diplomyelia) (Case 4: 9-day-old, male, Holstein Friesian). **a**-**e**: MRI T2weighted (T2w) of calve with diplomyelia. **f**-**g**: MRI T1weighted (T1w) of calve with diplomyelia. **a**, **f**: Sagittal image of the spinal cord from L3 to the sacrum. At the level of L4 the spinal cord contains a structure filled with cerebrospinal-like fluid (asterisk *) in the dorsal funiculi. A slight dorsal narrowing (hash #) of the vertebral canal causes a slight compression of the spinal cord. Blue lines indicate the level of the transversal images shown below (B-E). **b**-**e**, **g**: transverse sections of the spinal cord at the level of L4-L6. **b**: Ventral aspect of cranial part of the fluid-filled structure (asterisk *) two small hyperintense (B-E), respectively hypointense (G) dots indicate the presence of two central canals (arrows ↑). **c**: The caudal end the fluid filled structure (asterisk *) is divided by a midline septum. Ventral of the structure the spinal cord shows a partial division and two central canals on each half are visible (arrows ↑). The left one seems slightly delated. **d**: The division of the spinal cord is more obvious. Both central canals are clearly visible (arrows ↑). **e**: A slight right dorsal narrowing (hash #) of the vertebral canal causes a slight compression of the spinal cord. Both central canals are still appreciable (arrows ↑). **i**-**ii**: MRI T2w of a healthy calf. **i**: transverse sections of the spinal cord at the level of L3. **ii:** Sagittal image of the spinal cord from L3 to the sacrum

Images showed an intramedullary fluid filled cavity (hyperintense in T2w, hypointense in T1w) in the median of the dorsal half of the lumbar vertebral column at the level of L4 (Fig. 1, A, B). The caudal part of the cyst was longitudinally divided by a thin septum (Fig. 1 C). Therefrom the spinal cord showed a peanut-like shape with a central canal in each half (Fig. 1d). A slight extradural dorsal narrowing of the vertebral canal at the level of the intervertebral disc space of L5-L6 by a soft tissue isointense structure did not cause significant spinal cord compression (Fig. 1e). The brain and the remaining spinal cord were unremarkable.

### Euthanasia of the animals

Once the diagnoses were performed, the animals 1, 2 and 3 were euthanized with T61 (tetracainehydrochloride 5000 mg, Intervet, Germany; 5 ml/50 kg BW; i.v.) after induction of narcosis with Xylavet® (xylazine hydrochloride, 20 mg/ml; CP-Pharma Handels GmbH, Burgdorf, Germany; 0.5 mg/kg, i.v.) in combination with Ursotamin® (ketaminehydrochloride 100 mg/ml; Serumwerk Bernburg AG, Bernburg, Germany; 2 mg/kg, i.v.). Case 4 was euthanized with Release® (pentobarbital-natrium 300 mg/ml; WDT Garbsen, Germany; 300 mg/10 kg BW; i.v.).

### Pedigree analysis

**Fig. 2:**
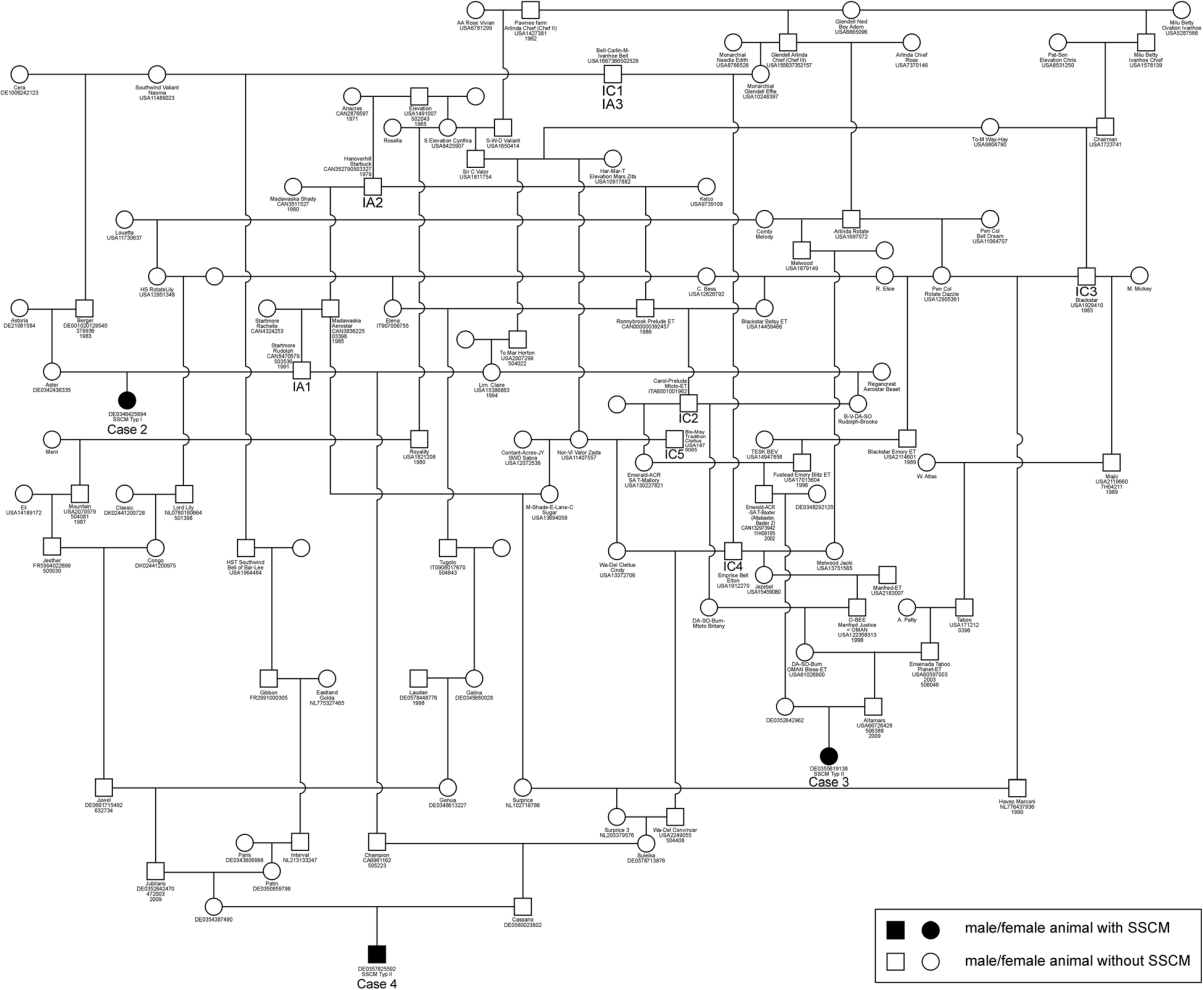
Pedigree of the three SSCM calves (cases 2–4). The sire of case 2 (IA1) is the paternal grand-grandsire of case 4 and an ancestor of case 3 in generation six. Further ancestors corresponding together in the pedigree of the three cases are marked IA1-IA3. The main contributors to the inbreeding coefficients are IC1-IC5

Using the program OPTI-MATE, version 4.0 [21], pedigrees of cases 2–4 were analyzed for relationship and inbreeding coefficients as well as the genetic contributions of ancestors to the cases. For case 1, a pedigree could not be obtained because the dairy farm was given up and the former owner could not be ascertained. Common ancestors for cases 2–4 were found in generation six and later generations (Fig. 2). Inbreeding coefficients of cases 2–4 were 0.098, 1.367 and 0.537% with completeness indices of 39.5, 51.2 and 65.2%, respectively. The main contributors to the inbreeding coefficients were the sire IC1 for case 2, sires IC2 and IC3 for case 3, and sires IC4 and IC5 for case 4. The relationship coefficients among cases 2 and 3, 2 and 4 as well as 3 and 4 were 2.34, 7.62 and 1.71% with completeness indices of 51.2, 55.0 and 64.3%, respectively. The relationship coefficients among IC1 and IC4, IC2 and IC3, IC1 and IC2 as well as IC3 and IC5 were 50, 31.8, 7 and 8% with completeness indices between 32 and 75%. The most important common ancestors (IA) with a high relationship to the three cases were sires IA1-IA3.

### Pathological findings of the central nervous system (CNS)

**Case 1:** SSCM type II (diplomyelia) was characterized by duplication of spinal cord tissue within common leptomeninges and dura extending from the 2nd to 3rd lumbar vertebra. Malformation was further associated with tubular cavitation of CNS tissue (syringomyelia) in the dorsal white matter.

**Case 2:** SSCM type I (diastematomyelia) with complete duplication of spinal cord tissue and separate meningeal coverings was present within the lumbar canal. In addition, a meningocele was found within the ventral sulcus.

**Case 3:** SSCM type II (diplomyelia) was observed within the lumbar and adjacent sacral vertebral canal.

**Fig. 3:**
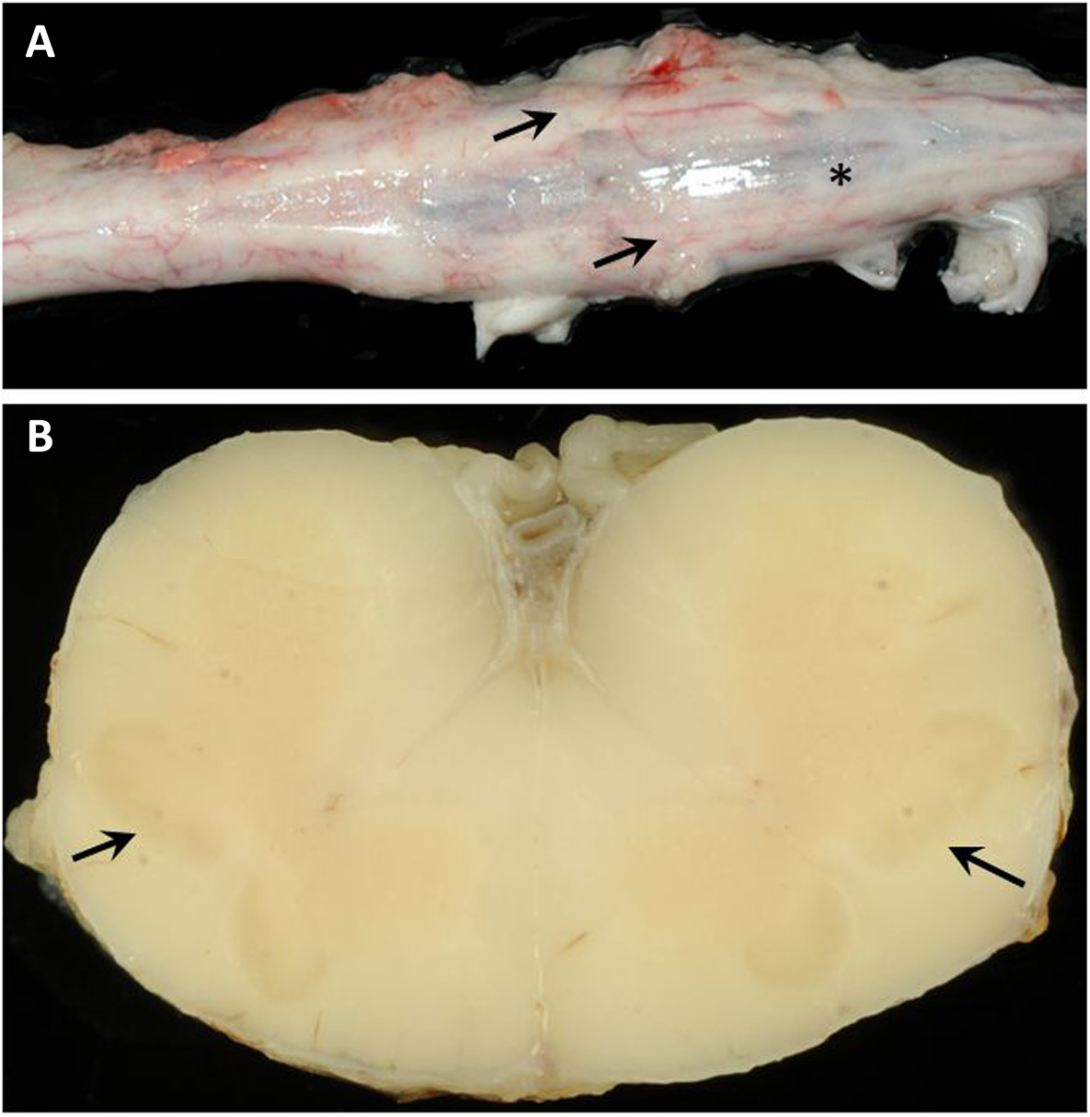
**a**: Necropsy findings of a calf with SSCM type II (diplomyelia) (case 4: 9-day-old, male, Holstein Friesian). **a**: Dorsal view of the spinal cord with divided lumbosacral intumescence (arrows) and dorsal segment with hollow cavity (asterisk). **b**: Transversal section of the spinal cord. Note duplication of the grey matter (arrows)

**Fig. 4:**
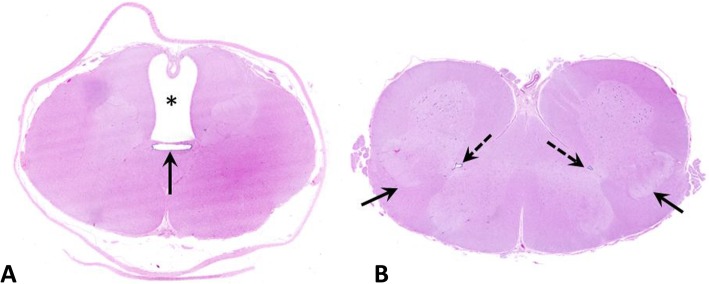
Transversal sections of the spinal cord of a calf with diplomyelia (case 4: 9-day-old, male, Holstein Friesian). **a**: tubular cavitation (syringomyelia) of the withe matter (asterisk) and dilated central canal (arrow). **b**: Duplication of the grey matter (solid arrows) with presence of two central canals (dashed arrows)

**Case 4:** SSCM type II (diplomyelia) (Fig. 3a and b) with dilatation of the central canal (hydromyelia) and syringomyelia within the dorsal white matter was present in the lumbar spinal cord (Fig. 4a and b).

## Discussion and conclusion

The here presented four cases of SSCM were referred to the Clinic because of unspecific signs of difficulty or inability to stand. Several illnesses or metabolic disorders may cause such disturbances in locomotion in the first weeks of life such as neonatal diarrhea, cerebro-cortical necrosis secondary to vitamin B1 deficiency, ruminal acidosis of the milk-fed calf, nerve lesions secondary to dystocic calvings or intense obstetrical assistance, bone fracture or severe trauma. If those affections have been ruled out during the clinical examination, a thorough neurologic clinical examination is needed for the diagnostic approach. The differential diagnosis of animals presenting deficits in the neurological examination of the spinal cord should consistently include conditions such as spina bifida [18, 22], segmental dysgenesis [23] and spinal stenosis [24] but also SSCM, even though they are sporadic diseases in bovine.

However, the clinical examination and diagnosis of pathologies of spinal cord conditions can be extremely difficult since several factors related to the lesion influence the clinical signs, including the type of lesion, location and extent. The neuroanatomical localization and extension of the lesion are responsible for the neurological deficits of the limbs and the trunk. In the present report the pelvic limbs displayed more profound neurological deficits than the thoracic ones in case 3 and 4. This is consistent with the histopathological observations. The cervical intumescence or its proximities were intact in all cases of this report. According to Vitelozzi et al. [8] the localization of SSCM is more common in the lumbosacral segments than in the thoracic ones. Lesions in the cervical segments are rare according to these authors. Nonetheless, SSCM in the cervical intumescence [7], cervico-thoracic [10] and thoraco-lumbar [16] segments have also been described in bovines. The differentiation between type I or II of SSCM attending to clinical signs is very difficult, if not impossible [25]. Imaging diagnostic tools such as ultrasound [15] or MRI [7] may be decisive for the diagnosis in vivo but require experience and ability (ultrasound) or are expensive and not indicated for the ambulatory clinic (MRI). In the present report, the diagnosis of SSCM in case 4 was made by MRI. Additionally, a type II SSCM was suspected because of the presence of a thin septum dividing the neural cord into two similar halves, each with a central canal. The clinical diagnosis was confirmed by histopathology.

According to Pang et al. [4] and Dias and Pang [6] the SSCM can be classified into two groups attending to the composition of the dural sheath and the implicated mesenchymal tissue. Thus, type I consists of two hemicords with dural tubes and separation by a dura-sheathed rigid median septum. In case of type II SSCM the hemicords are encased within a single dural sac and only separated by a nonrigid, fibrous median band [7]. In cases 1, 3 and 4 the spinal cord was duplicated within common leptomeninges whereas in case 2 the spinal cord was complete duplicated and separate meningeal coverings were present. Hence, cases 1, 3 and 4 were histologically diagnosed as diplomyelia or SSCM type II and case 2 as diastematomyelia or SSCM type I.

To explain the differences between Type I and II embryological aspects have to be included, which are a subject of controversial discussion. The most accepted theory, original from Bell’s work [26], proposed by Bremer’s [27] and updated by Pang et al. [4], is based on the existence and persistence of an abnormal accessory neurenteric canal (ANC) [7] as a result of adhesions between the ecto- and endoderm. Chronologically, in the first weeks of gestation there is a primitive neurenteric canal (PNC), which transitorily connects the yolk sac, with endodermic origin, and the amnion, with ectodermic origin [3, 25]. Once the PNC is closed, a new aberrant neuro-ectodermic communication, the ANC [7], may develop. The persistence of the intermediate part of the ANC causes the division of the notochord and the neural placode [27]. The dissection of the notochord interferes with the formation of vertebral bodies. If the neural placode is bisected into two hemi-placodes, two hemicords will ensue, causing a split cord malformation. At this point, two structures will determine if a SSCM Type I or II, or both concomitantly in different loci of the cord (namely *composite* lesions) will develop: the mesenchyme surrounding the ANC, precursor of the dural sac, and the primitive meningeal cells (meninx primitiva). Simplified, if the mesenchyme surrounding the ANC incorporates primitive meningeal cells, pluripotential, and are therefore able to transform into bone, cartilage, and fibrous tissues, a SSCM type I results. However, if the mesenchyme of the ANC has not incorporated meninx primitiva, then the formation of the dural sac occurs normally. The ANC either disappears or is transformed into an intradural non-rigid spur situated between the two hemicords, constituting a SSCM Type II [3]. Probably, the persistence of the ANC interferes with the neurulation process, leading to probable myelodysplastic lesions such as paramedian nerve roots, myelomeningocele manqué, and centromedian vascular structures, mono- or bilaterally. These lesions may act as confounders since they may be present in both cases of SSCM [4]. Indeed, in cases 1, 2, and 4 a syringomyelia, a meningocele and a hydromyelia together with syringomyelia were stated, respectively.

Attending to the classification of Bhidayasiri [20], case 4 showed a low frequency resting tremor. A similar kind of tremor has already been described in the literature in Holstein Friesian cattle [28]. In that work, the tremor was attributed to microscopic degenerative lesions in the white substance of spinal cord and brain, more concretely demyelinitation in both ventral and dorsal radicles. In case 4, myelin sheaths with myelophages at the cervical marrow were detected. The presence of myelophages indicates demyelinitation and may be associated to the resting tremor observed in our patient. Furthermore, the Wallerian degeneration involves necrosis and demyelinitation of motoneurons and is known to be related to tremor in horned Hereford calves diagnosed with “Shaker-Calf” syndrome [29]. However, in case 4, necrosis was not observed in the central nervous system. The calf in case 4 also presented a deficiency of thiamine-monophosphate and thiamine-diphosphate but normal values of total thiamine. Polioencephalomalacia or cerebro-cortical necrosis due to thiamine deficiency was discarded as the main cause of the central nervous system alterations of this patient. It is possible that low levels of thiamine-diphosphate in blood and concomitantly in brain and spinal cord parenchyma could have exacerbated the neurological findings, but this fact remains high speculative since neuronal necrosis was not observed histologically. Further abnormal blood parameters (leukocytosis, neutrophilia, hypoproteinemia and hypoalbuminemia) are very likely due to an insufficient colostrum intake and secondary inflammatory processes (acute bronchopneumonia, omphalophlebitis and bursitis precarpalis).

Pedigree analysis revealed a familiar relationship of cases 2–4 and suggested this malformation to be an inherited disorder. The pedigree data support the hypothesis of a recessive mode of inheritance due to common ancestors resulting in homozygosity of partial segments of the genome. A dominant Mendelian or X-linked mode of inheritance can be excluded because the parents and grandparents did not present this malformation and female calves were also affected. In addition, a germline mutation cannot be ruled out with a possible starting point in sire IA1, because he is a direct ancestor of the three cases. However, this is quite unlikely because this bull is a commonly used AI bull and, in such cases, the ratio of defective offspring may be very high. Incomplete duplications would be also conceivable.

Accumulation of recessive mutations is enhanced through inbreeding. In the cases under analysis, we were able to calculate inbreeding coefficients even if pedigrees were not fully complete. Thus, we expect that inbreeding coefficients are slightly underestimated. Familiar relationships are a prerequisite for supposing a hereditary condition what was shown for the cases herein analysed. In Holsteins, inbreeding was found in about 70% of 74,946 animals using data from three German commercial dairy farms [30]. The average inbreeding coefficient of these animals was 2.255% with a pedigree completeness of 48%. A significant effect of a higher degree of inbreeding on the increase of stillbirths and lower birth weights in calves was shown in this study and this finding may be indicative that the risk of homozygous recessive mutations with deleterious effects is larger in inbred Holsteins. Thus, we assume that in our cases defective alleles accumulated through inbreeding on the most important sires and made this rare malformation evident. Calves with a similar pedigree but not affected did not receive all these defective alleles due to random segregation of alleles. The more defective alleles are required for expression of a specific malformation the lower is the frequency of the malformation in a sample. Possibly more than one locus is involved in the development of SSCM.

In conclusion, 4 cases of SSCM were described; two of them included the findings of the clinical examination and in one case SSCM was diagnosed by MRI. Although SSCM are sporadic diseases in bovine, they should be considered as differential diagnosis of animals presenting deficits in the neurological examination of the spinal cord. Therefore, we firmly advise practitioners to execute the neurological examination of calves to exclude congenital pathologies of the central nervous system. This is the first description of familiar cases of SSCM in Holstein cattle with first possible considerations of the mode of inheritance. Further investigations with more SSCM Typ I and Typ II cases are warranted in order to identify responsible mutations and to analyse the possible mechanism for their development.

## Data Availability

The datasets used and analysed during the current study are available from the corresponding author on reasonable request.

## Abbreviations

°C: grad Celsius
ANC: accessory neurenteric canal
BVD: bovine viral diarrhea
BW: body weight
cm: centimeter
CNS: central nervous system
ELISA: Enzyme-linked immunosorbent assay
Fig.: Fig.
I.U.: international units
IA: important ancestors
IBR: infectious bovine rhinotracheitis
kg: Kilogramm
L: Liter
L4–6: lumbar vertebras 4 to 6
L6-S1: lumbosacral junction
mg: milligram
mL: milliliter
MRI: magnetic resonance imaging
PCR: polymerase chain reaction
PNC: primitive neurenteric canal
s.c.: subcutaneously
SBV: Schmallenberg virus
SSCM: split spinal cord malformation
T1w: longitudinal relaxation time weighted
T2w: transverse relaxation time weighted
μg: microgram
μL: microliter
